# The Effect of Alpha-Lipoic Acid on Diabetic Peripheral Neuropathy and the Upcoming Depressive Disorders of Type II Diabetics

**DOI:** 10.7759/cureus.12773

**Published:** 2021-01-18

**Authors:** Dimitrios T Karalis, Tilemachos Karalis, Stergios Karalis, Angeliki S Kleisiari, Foteini Malakoudi, Konstantina Eleni V Maimari

**Affiliations:** 1 Nutrition and Dietetics, University of Thessaly, Volos, GRC; 2 Obstetrics and Gynecology, General Hospital of Trikala, Trikala, GRC; 3 Internal Medicine, General Hospital of Trikala, Trikala, GRC

**Keywords:** alpha lipoic acid, diabetes type 2, type ii diabetes, diabetic complications, diabetic neuropathy, depression

## Abstract

Introduction

Peripheral neuropathy is one of the possible complications of diabetes. Alpha-lipoic acid (a-lipoic acid or ALA) is a powerful antioxidant cofactor synthesized in mitochondria that could help stimulate nerves and regenerate nerve fibers, thus preventing disease progression. Moreover, the possible feeling of oppression from the lifestyle changes needed to avoid the complications of diabetes may contribute to the development of depressive symptoms. ALA increases insulin sensitivity, which could increase serotonin synthesis and thus reduce the manifestations of depressive disorder.

Aim

The aim of this study is to investigate the therapeutic effect after oral administration of a-lipoic acid in patients with type II diabetes mellitus, regarding the possibility of developing peripheral neuropathy and the possibility of developing depressive disorder due to the existence of diabetes type II.

Methods

The study sample consisted of 148 Greek patients, type II diabetics, 68 men and 80 women, aged 50-75 years. All of them were non-smokers and did not consume alcohol. Their treatment was a combination of gliclazide, sodium-glucose-linked transporter 2 (SGLT-2) inhibitors, metformin, and glucagon-like peptide 1 (GLP-1) analogs. None of them were under insulin administration. Any other treatment received chronically from the patients for other comorbidities was not altered or paused. All patients were in regular monitoring of renal, hepatic, and ocular function, which was normal. Patients were monitored with a balanced diet, based on equivalents, in order to maintain an almost constant body mass index (BMI). All were given one tablet of 600 mg a-lipoic acid, two hours before a meal, for eight months, and the incidence of developing peripheral neuropathy and depressive disorder was assessed, using the Subjective Peripheral Neuropathy Screen Questionnaire (SPNSQ) and Beck Depression Inventory (BDI) questionnaire.

Results

ALA administration after both four and eight months resulted in statistically significant results and, specifically, the peripheral neuropathy development mean score was reduced by 4.79 at four months and 6.22 after eight months. Concerning the incidence of depressive disorder, an average decrease of 4.43 in the related depression score was observed at the four-month milestone and 7.56 at eight months, both statistically significant.

Conclusion

A-lipoic acid is a powerful antioxidant and, when used with conventional treatment, has shown to significantly decrease the incidence of depression and peripheral neuropathy in patients with type 2 diabetes mellitus.

## Introduction

Αlpha-lipoic acid (a-lipoic acid or ALA) is a cofactor found under normal conditions in most eukaryotic and prokaryotic organisms. It is synthesized in the mitochondria. It is essential for the proper functioning of several enzymes that play a key role in oxidative metabolism [1-2]. In particular, it is a powerful antioxidant that is soluble in both water and fat; thus, it is easily absorbed after oral administration [3-6].

It was first isolated in 1950 by Reed and colleagues from bovine liver as a catalyst associated with pyruvate dehydrogenase [7]. In 1992, Reed and Cronan for the first time identified the DNA sequence of two genes, lipA and lipB, which were involved in the biosynthesis and metabolism of ALA as a part of the Escherichia coli genome [8]. Subsequent analyses of the Escherichia coli genome developed further knowledge of the pathways of the synthesis, adhesion, and functioning of ALA [9].

The human body cannot synthesize sufficient amounts of ALA to meet its needs. Thus, it is necessary to obtain it from dietary sources [10]. The bioavailability of ALA decreases significantly with food intake. It is, therefore, recommended to administer it at least two hours before eating; if this is not possible, at least 30 minutes post-meal [11]. This is mainly because ALA, being a weak acid, needs the acidic pH of the stomach to be absorbed readily. Food interferes and competes with the absorption [11-13].

One of the complications of diabetes is peripheral neuropathy. It is an aesthetic-motor polyneuropathy with progressive severity of symptoms that results in functional decline and reduced quality of life [14-15]. As a powerful antioxidant factor, ALA helps stimulate nerves and regenerate nerve fibers, thus preventing the progression of diabetic neuropathy and helping alleviate its symptoms [16-17].

Depression has been related to altered levels of neurotransmitters in the human brain, in particular, serotonin [18-20]. Serotonin and other monoamine neurotransmitters are synthesized from the amino acid precursor tryptophan [21]. Insulin as a whole increases the influx of tryptophan in the brain, resulting in the synthesis of serotonin [22]. In diabetic patients, the reduced secretion or reduced activity of insulin relates to the reduction of the ratio of tryptophan to other plasma amino acids, resulting in a reduced availability of tryptophan in the brain and, therefore, a decrease in serotonin concentration [23]. Thus, increasing insulin sensitivity may lead to increased tryptophan availability and, therefore, an increase in the production of serotonin and other monoamines in the brain [22]. Due to its ability to increase insulin sensitivity, ALA could help improve the influx of tryptophan into the brain, causing an increase in serotonin synthesis and resulting in a reduction in depression [24].

 In the present study, we will try to investigate the therapeutic effect of oral a-lipoic acid in patients with type II diabetes, in terms of the incidence of peripheral neuropathy and depressive disorder.

## Materials and methods

The study sample consisted of 148 patients, 68 men and 80 women in the age group of 50-75 years. The study included patients in the healthy weight range (body mass index (BMI) 18-25 kg/m^2^), overweight (BMI 25-29.9 kg/m^2^), and obese patients (BMI >30 kg/m^2^). All subjects in the study declared that they were non-smokers and did not consume alcohol. Inclusion criteria in the study were the existence of type II diabetes (based on the diagnostic criteria set by the World Health Organization), not taking insulin for glycemic control, and the absence of mental disabilities in order to achieve a correct subjective assessment of the complications of diabetes mellitus. All patients received 600 mg of a-lipoic acid daily, in tablet form, instructed to take it at least two hours before meals, for a period of eight months, and the possibility of peripheral neuropathy and depressive disorder was evaluated. The diabetic management of each patient remained the same as before the study. Patients were on a combination of various drugs, including gliclazide (sulfonylurea), sodium-glucose linked transporter 2 (SGLT-2) inhibitors, metformin, and glucagon-like peptide 1 (GLP-1) analogs. Patients on antihypertensive and lipid-lowering drugs continued their treatments. In addition, all volunteers had good renal and hepatic function and were under medical supervision for the early diagnosis and treatment of possible health problems. Finally, all participants were informed about the aims of the study, as well as about the process of measurements, evaluations using questionnaires, and the medical examinations to which they would be submitted and gave their approval for their participation, through written consent (according to the protocols of the Research Ethics and Conduct Committee).

The height of the participants was measured, once, using a built-in stature meter in an upright position, without shoes, and with an accuracy of 0.5 cm. Weight was measured in light clothing and without shoes, on an electronic precision balance, and the measurements were rounded to the nearest hundred grams. The scales were adjusted and checked before and after each weighing. The body mass index (BMI) was then calculated as the quotient of weight (measured in kilograms, kg) to the square of height (measured in meters, m).

Two questionnaires were selected as the most appropriate tools for the evaluation of possible peripheral neuropathy and depression. The Subjective Peripheral Neuropathy Screen Questionnaire (SPNSQ) was selected to assess the likelihood of developing peripheral neuropathy, which consists of 15 "yes/no" questions about the symptoms of the neuropathy, and the score is calculated by summation of the "yes" answers. The score ranges from "0" (no neuropathic symptoms) to "15" (the most severe neuropathic symptoms). The “Aaron T. Beck questionnaire” (Beck Depression Inventory - BDI), which is a self-assessment questionnaire consisting of 21 multiple-choice questions, was selected to assess the presence of depression. It is a widely used instrument for measuring the severity of depression. Each response is scored on a scale from 0 to 3. Higher overall scores indicate more severe depressive symptoms. The overall score is evaluated as follows: 0-13: minimal depression, 14-19: mild depression, 20-28: moderate depression, and 29-63: severe depression.

The diet of each patient was monitored. They were given a balanced diet in order to maintain a relatively stable body mass index during the eight months of the study. In addition, they were given general dietary instructions based on the special needs of the diabetic population.

The analysis of the research results was done with the Statistical Package for the Social Sciences (SPSS) (Version 25, IBM Corporation, Armonk, NY). The research sample consisted of 148 patients (N = 148). For the correlation test between the two periods, from zero to four months and from zero to eight months, Pearson's linear coefficient was applied to the data with values being significant at <0.05 (correlation positive at p<0.05). Results were evaluated in the form of tables and graphs.

## Results

**Figure 1:**
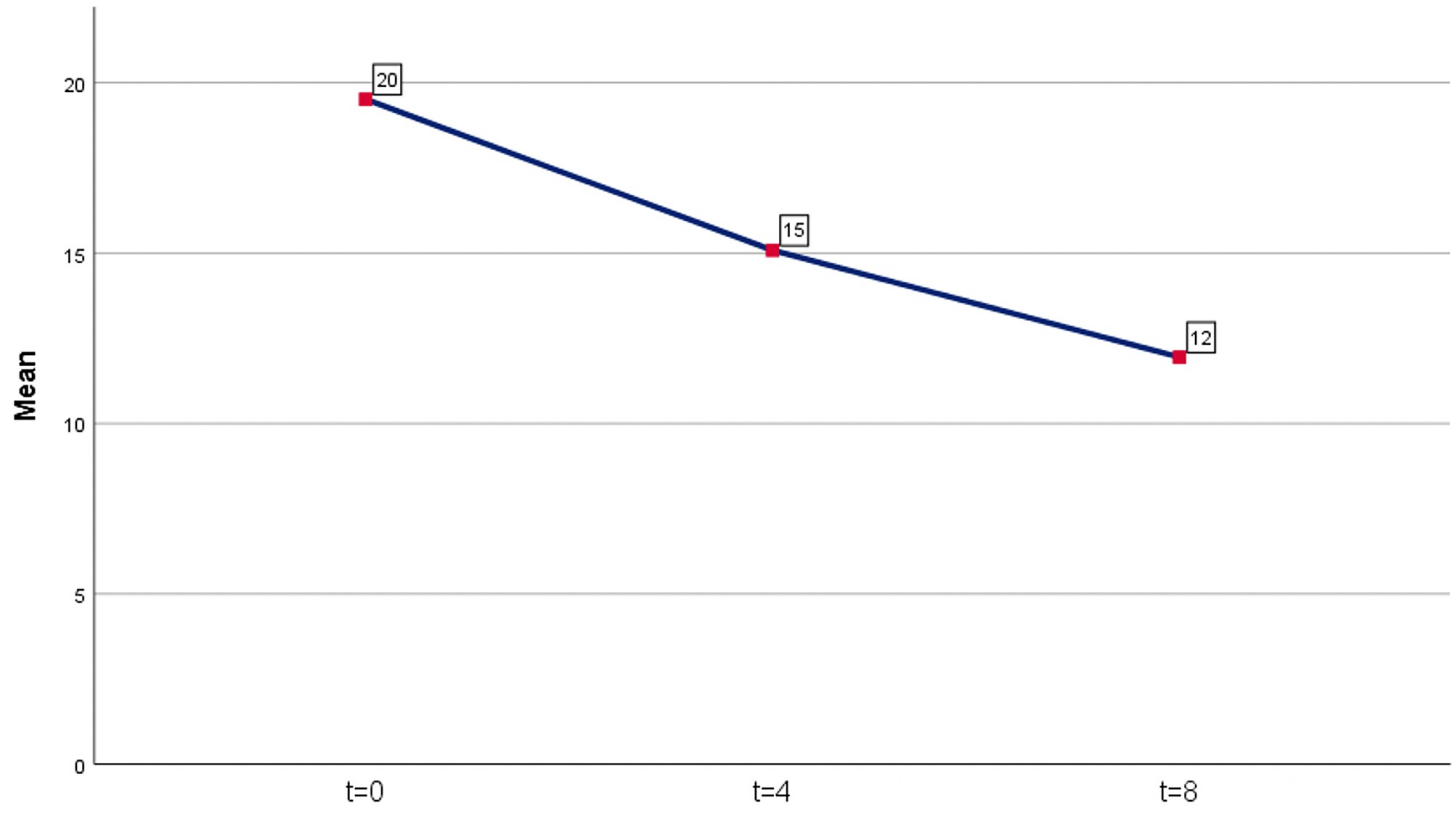
Average BDI levels (rounded, no decimals) BDI: Beck Depression Inventory

A statistically significant decrease in the score points of the BDI questionnaire was observed in both time periods. For the first period (four months after the administration of ALA), an average decrease of 4.43 points was recorded (mean reduction = 4.43, percentage 22.7%). The results were statistically significant (p = 0.00001 <0.05). For the second period (eight months after the administration of ALA), an average decrease of 7.56 points was recorded as compared to day 0 (mean reduction = 7.56, percentage 38.7%). The results were also statistically significant (p = 0.00001 <0.05). The results are shown in Table 1 and Figure 1.

**Table 1:** Descriptive statistical measures for BDI questionnaire score (zero, four, and eight months) BDI: Beck Depression Inventory

	N	Minimum Value	Maximum Value	Mean Value	Std. Deviation
BDI before	148	5	35	19.51	5,920
BDI 4 months later	148	2	32	15.08	6,214
BDI 8 months later	148	1	33	11.95	6,384
Valid N (listwise)	148				

**Figure 2:**
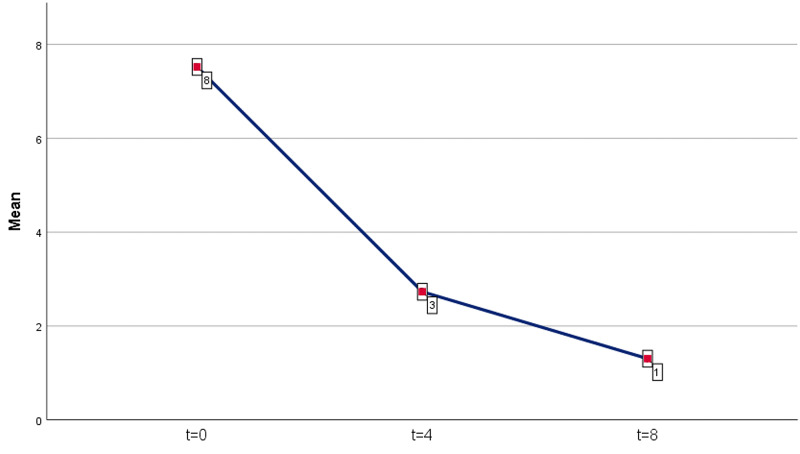
SPNSQ level average (rounded, no decimals) SPNSQ: Subjective Peripheral Neuropathy Screen Questionnaire

A statistically significant decrease in the score points of the SPNSQ questionnaire was observed in both time periods. For the first period (four months after the administration of ALA), an average decrease of 4.79 points was recorded (mean reduction = 4.79, percentage 63.7%). The results were statistically significant (p = 0.00001 <0.05). For the second period (eight months after the administration of ALA), an average decrease of 6.22 points as compared to day 0 was recorded (mean reduction = 6.22, percentage 82.7%). The results were also statistically significant (p = 0.00001 <0.05). The results are shown in Table 2 and Figure 2.

**Table 2:** Descriptive statistical measures for the SPNSQ score (zero, four, and eight months) SPNSQ: Subjective Peripheral Neuropathy Screen Questionnaire

	N	Minimum Value	Maximum Value	Mean Value	Std. Deviation
SPNSQ before	148	3	15	7.52	2,737
SPNSQ 4 months later	148	0	12	2.73	2,032
SPNSQ 8 months later	148	0	6	1.30	1,249
Valid N (listwise)	148				

## Discussion

This study investigated the therapeutic effect of orally administered 600 mg a-lipoic acid on depression and peripheral neuropathy related to diabetes. It concluded that there is a statistically significant improvement in the prevalence of depression and peripheral neuropathy with the administration of 600 mg a-lipoic acid daily in diabetics.

The positive effect of a-lipoic acid has been well-researched and proven in peripheral neuropathy. A study conducted in 2002 evaluating the safety and efficacy of a-lipoic acid in the total symptom score (TSS), a measure of positive neuropathic sensory symptoms, demonstrated an improvement of the TSS score of the ALA receiving group from the baseline by an average of 5.7 points as compared to the group receiving placebo that had an average of 1.8 points [25]. Another study that evaluated the progression of diabetic neuropathy after the long-term administration of ALA during a period of four years demonstrated that participants taking ALA showed stabilization of the disease, in contrast with the conventional therapies for the treatment of symptoms, such as duloxetine, pregabalin, and gabapentin, which did not appear to stop the progression of the condition [26]. Finally, a meta-analysis of four double-blind placebo-controlled studies involving more than 1200 patients showed that a-lipoic acid at a dose of 600 mg daily significantly improved the clinical picture of patients with diabetic neuropathy [27].

We found significant improvement in the depressive symptoms of our patients, which is a finding supported by previous literature. A review study evaluating the potential of ALA administration in various psychiatric and neurological diseases and symptoms concluded that ALA is a promising adjuvant for treating depression as demonstrated by the hormonal fluctuations in the prefrontal cortex of mice models [28]. Finally, a study of mice models with pharmaceutically induced depression demonstrated that the oral administration of ALA through a feeding tube increased the levels of brain-derived neurotrophic factor (BDNF), similar to anti-depressants, supporting that ALA administration could have an anti-depression potential [29].

## Conclusions

In conclusion, the present study supports the existing research claims for the significant benefits of a-lipoic acid administration in reducing the likelihood of developing peripheral neuropathy in diabetic patients. In addition, the results of the study show that the daily administration of a-lipoic acid results in a significant reduction in depressive symptoms. Further clinical trials are needed to demonstrate whether the benefits of a-lipoic acid supplementation can be long term in diabetic populations as well as in individuals with diseases strongly associated with the presence of oxidative stress in the human body.
